# Monstrosities

**Published:** 1835

**Authors:** 


### REVIEWS AND BIOGRAPHICAL NOTICES.

---

### Art. VI.—Monstrosities.

*Lesions of Nutrition depending on the irregular Arrangement and Distribution of the anatomical Elements, of which the Solids of the Body are, in their natural States, composed. Andral's Pathological Anatomy, Vol. I. p.* 69—122.

By the Assistant Editor.

Although Pathological Anatomy has been cultivated with the utmost zeal, on the continent of Europe, during the last quarter of a century, the study may be said to be still in its infancy in the American schools. Indeed, there is but one in the Union, in which lectures are especially delivered on that branch of Medical Science, although it is every where acknowledged to be the basis of a sound and well regulated professional education. The physician, who is ignorant of the various alterations of structure which it reveals, is unfit for the duties assigned him, although he may carry with him the lettered parchment, together with all the plumage with which it is decked, by the most refined modern taste. It is the study of this department alone, which enables him to detect the various morbid derangements of any or of all the organs; to trace their origin and explain the processes by which they pass through their various stages, and to note their mutual relations, and the order of succession in which they attack the different tissues of the body. But why need we urge the study of this branch of our profession? It will at once be seen, and appreciated, by every lover of truth and philosophy.

Until the present century the history of morbid anatomy, was confined to isolated facts, widely separated from each other, aad mostly buried beneath the general rubbish of medical periodicals. It is true the names of Bailie, Bichat, Corvisart, Laennec, and others, have been intimately associated, for the last forty years, with this department of general pathology; but their observations, however valuable, were confined to single lesions, or to the morbid structure of some particular part of the human body. Dr. Bailie is the only one who presented the profession with an extended view of anatomical lesions. The investigations of Laennec, though sufficient to wrest his name from the ruins of time, were entirely confined to the chest. Indeed he has left but little to be accomplished by any of his successors in relation to the diseases of the thoracic vicera. Louis, it is true, has done much, in his late researches in phthisis, to exhibit the true nature of this destructive malady, but still he has only completed and decorated the edifice, which may almost be said to have been planned and reared by Bailie and Laennec. Our author, Andral, has not stood aloof from the unfortunate consumptive. He, too, has thrown much light upon the tubercular formation, and proved to the profession at large, the fallacy of the exclusive solidism of Broussais.

But he has not only enriched and extended the works of his predecessors; he has also added much to pathological anatomy from his own observation; the whole of which he has arranged into a beautiful and philosophical system. In fact he has done for pathological, what Bichat did, a few years before, for general anatomy.

It is not our intention to present the reader at this late period, with an extended review of the work of Andral. To do this would require more time than we are able to bestow on the subject. Indeed the entire work should be in the hands of every physician, who should study it with as much attention as the most ardent Christian does his Bible. We shall only direct the attention of our readers to that portion of the chapter, on the lesions of nutrition, which treats of the congenital malformations usually termed monstrosities.

Until recently, this subject has been involved in much obscurity. The older writers applied the term monstrosity only to the most hideous congenital aberration. It is to the industry and perseverance of later anatomists that we are indebted for a philosophical view of the subject. Even as late as the past century, some of the European Journals abounded with the most fanciful drawings, and vague descriptions of monsters varying in form and size, but always resembling, to some extent, certain animals which had alarmed the mother during her pregnancy. These imaginary beings, however, not only resembled the form of certain animals, but they also frequently combined all that was uncouth and terrific in any number of animals whatever. It is well known that many, even at the present day, attribute much, in the growth of the fœtus, to the imagination of the mother. This idea is not confined entirely to the vulgar. Some of the physicians of the present day, especially among the Germans, seem still to adhere to this ancient popular notion. Something for which the mother has longed without being able to obtain, or the sudden approach of some frightful aminal or other object of terror, may either mark or vary the form of the unborn child. This impression was extensively prevalent in the early part of the eighteenth century, although Littre, at a much earlier period, advanced the idea that certain kinds of monstrosities consisted in an arrest or suspension of nutrition at various periods of utero-gestation. This assertion, however, seems to have been entirely overlooked by medical writers, until the time of M. Geoffrey, St. Hillaire, who, together with his cotemporaries, seized upon it, and developed and extended it, until they erected on the ruins of the former absurd hypotheses, a most beautiful and philosophical system. But an arrest or suspension of nutrition, will not always explain the growth of certain monsters. In many cases the formative process, not only seems to be increased, diminished, or arrested, but it is also perverted, or *sent estray.* The organs or tissues are frequently transposed, or placed in situations more or less distant from the places they usually occupy. Still nature seems to keep within certain bounds, for although she sometimes places the lungs in the abdomen, or the liver in the thorax, the brain has never been found in the pelvis, nor the bladder or uterus in the cranium. But let our author speak for himself in relation to such lesions. On page 72 he observes:

"The development of the fœtus may be modified in various ways: sometimes the formative process, or nisus formativus, as it has been termed, possesses less energy than natural, and the development of the organs is in consequence suspended; in which case they are found either imperfectly formed, or altogether deficient: sometimes, on the other hand, this force seems to acquire an access of energy, and then, there is a corresponding excess of development, and the organs exceed their natural limits either in size, or number. In other cases again the development cannot be properly said to be either excessive or defective; but the formative process appears to have been simply perverted, thus producing various modifications in the direction, and situation of the organs. We have examples of this in the general transposition of the viscera, and in certain varieties in the origin of the principle arteries."

He frequently refers, in various parts of his work, to this perversion of nutrition, in order to explain many facts in pathology, hitherto but little understood. The melanotic changes, together with the osseous and cartilaginous transformations, are of this class.

Meckel, in his remarks on monstrosities, is disposed to think that such creations may vary in form, from the slightest departure from the usual shape, to the most unnatural appearance, and that all the intermediate grades may be the results of a collection of deformed parts from other species, so that a distinct kingdom of monsters might be given to the profession. St. Hillaire, however, was inclined to believe that every monster constituted in itself a kind of new creation or distinct species of animal. The remarks already quoted, will show that our author does not think with either of these able physiologists. He correctly views every monster as a part of the same genus and species with its parents; the alteration in form being only the result of a vice of nutrition.

It has been said by many physiologists, that man, in his passage from the embryotic state to maturity, exhibits at some period or other, the form of most, if not all, of the inferior animals. If this be the case, the position of our author will readily explain, as he centends, the resemblance of many monsters to the lower orders of animals; while it will, at the same time, show the reason why the simple organization of the latter, can never reach the complicated structure of the former. Indeed it seems to us that man is only a collection, at various periods, of some portion of all that is below him in the scale of life, with the addition of mind and reason, which stamp him with the image of his CREATOR, and render him lord of the earth he inhabits. If this be the case any arrest of nutrition, in the superadded parts of the body, will leave him, to some extent, with the form of the animal, whose organization he most resembled when the lesion occurred. For example, in the language of our author, "the human brain arrested in its evolution, may present an appearance more or less analogous to the brain of fishes or reptiles, but the simple brain of these animals, can never attain the degree of complicated structure which the human brain presents."

A late writer, M. St. Hillaire, affirms that there is, in the animal economy, a kind of law of compensation, by which the excessive growth of one part is always accomplished at the expense of another. Thus, if either a hand or a foot is furnished with an additional finger, or toe, the opposite extremity will exhibit a corresponding deficiency. Our author has collected a number of cases which serve to illustrate and confirm the position of St. Hillaire, but such a law cannot be of universal application. We once knew a whole family who had additional fingers or toes on one or all of the extremities, and yet no one of them was defective in any part of the body. Meckel has carried this law of compensation still farther; for he affirms that the excess of growth in one child, is followed by a corresponding deficiency in those who follow it. This seems to us to be carrying the idea beyond all bounds. In the family above alluded to, there was almost always, an excess of nutrition with a multiplication of parts in every member of the family, while there was no defect in any.

Our author observes, that the organs supplied by the cerebro-spinal system of nerves, seldom present us with faults of conformation, while an irregular development is frequently the result of the agency of the great Sympathetic. The heart and lungs, for instance, are seldom increased or diminished in structure or number, while the urinary, genital, and vascular systems, often present us with irregularities of structure. Indeed, it is more than probable, that an excess or defect in the number of organs, is frequently if not always, the offspring of the distribution of arteries and nerves in the embryo, for wherever healthy blood is sent, accompanied with the necessary nervous stimulus, nutrition must be accomplished.

According to all that our author has been able to collect, female monsters are much more numerous than those of the male sex. This he accounts for in the following terms: p. 76.

"The excessive proportion of female monsters seems to depend on the circumstance, that during the earlier period of the evolution of the fœtus, as in the lowest classes of the animal kingdom, there is only one sex, namely, the female; so that in fact, when we say that the greater number of monsters are of the female sex, we only state in other terms, that in the greater proportion of monstrous formations, be their seat and nature what they may, the genital organs are arrested at an early period of their development."

He next proceeds to notice, the hereditary disposition there often seems to be to form monstrosities. This question we think is placed beyond a doubt. We can readily cite a case in point. A Mrs. —— the mother of a large family in West Virginia, had four fingers and two thumbs on one of her hands. The additional member appeared to be united to the hand only by the integuments, and hence it was incapable of any voluntary motion whatever. The daughter had two thumbs on each hand, both of which were furnished with muscles and placed, to some extent, under the control of the will. Some if not all of her sons, had a supernumerary toe on one or both of their feet, with the usual number of fingers on their hands. The children of the daughter, I have lately been informed, have generally six fingers, with occasionally an additional toe. In this case the aberration of structure was not only hereditary, but nature made the attempt to complete her work, by furnishing her new creation with muscles, nerves, etc. Meckel, Morand and, others, relate similar instances of hereditary aberration.

Our author next proceeds to the consideration of the three classes of monsters, and first of those which proceed from an imperfection of development. p. 78.

"This kind of monstrous formation does not occur with equal frequency in all the different organs. It may be laid down as a general law, that those organs are most subject to imperfect development, which are the latest in attaining their complete evolution; and that every imperfection of development an organ presents, corresponds exactly with the state of that organ in some of the stages of its development."

He next adduces a number of cases to illustrate the position he assumes in the above paragraph, all of which are conclusive and to the point. He says the intestinal tube is the first formed. It commences with the vesicula umbilicalis, and is gradually elongated into the great and small intestine. This portion has never been found wanting even in the most deformed fœtus. The same is the case with the urinary aparatus, as well the vascular and nervous systems.

The nerves, it seems, are formed before the spinal marrow, and the spinal marrow before the brain, so that one or both of these organs may be left out of the system, by an arrest of nutrition. The same is true of the heart, for even this instrument is not formed until a part of the arterial system is perfected. In relation to the growth of the body our author remarks. p. 81.

"If we direct our attention to the external surface of the body, and the principal regions of which it is composed, we shall find that those parts which are most frequently absent or imperfect, are precisely the same as are latest in attaining their natural development. For example, at the first period of its formation, the fœtus may almost be said to consist entirely of an abdomen; accordingly, this part, more or less completely formed, has never been altogether deficient in any monster: on the contrary, there are several cases on record, in which no trace could be discovered of either head, neck, thorax, or extremities: so that in fact nothing was to be seen but an abdomen, just as at the commencement of embryotic life. The external parts of generation do not appear until very late; and it is by no means uncommon, in the fœtus, arrived at its full term, to find these parts altogether deficient, or presenting some imperfection which constitutes their natural condition at an early period of their formation. The monstrous formations of the organs of sense are likewise subject to the same law. The eye which makes its appearance in the form of a black point, before any vistige of the external ear is perceptible, is not so often deficient as the ear. The eyelids, likewise, which are not developed until long after the ball of the eye, are more frequently absent or imperfect than the eye itself."

Our author next attempts to prove that many of the organs are independent of each other, or nearly so, during the early periods of their growth, and hence he infers that the nerves accomplish but little in the early stages of fœtal life. In this case we think he is rather too much inclined to generalize, for he afterwards acknowledges that notwithstanding the body may be developed without the brain, still if the cervical enlargement of the spinal cord does not exist, the upper extremities are constantly deficient. From this fact alone we must infer that the nervous influence controls, to some extent, the nutrition of many, if not all the organs.

He next proceeds to examine the connexion between the growth of the arteries and other parts of the body. Here he seems to think that the development of the organs does not depend upon the development of the arteries. St. Hillaire, however, advances a different position. The magnitude of the arteries is always in proportion to the size of the organs. Still it is difficult to distinguish, in the connexion of the various phenomena, the cause from the effect.

The accidental openings, or foramina, are next investigated by our able author. They are always situated near the median line of the body, and are consequently the result of a suspended nutrition. This is especially the case in the fissures found in the anterior portions of the thorax or abdomen. The spinal deficiency, usually termed spina bifida, may also be said to depend on the same cause. The hair lip, in all its forms, or even the entire absence of the lips, likewise invariably depend upon a suspended nutrition. It has been said by some that if the hair lip was the result of this cause the fissure would always be in the centre, which is not the fact.

This objection, however plausible at first view, is readily obviated, when we recollect that the upper lip is invariably formed, in the early periods of fœtal life, in four pieces. The lower lip is formed in two pieces, but they are always united at a very early period, hence a fissure here, is seldom found.

The genital organs may be extensively changed in their appearance, and the sex rendered extremely doubtful by an arrest of nutrition. It may be more or less extensive from the simple opening of the urethra of the male, immediately under the glans penis, to the entire division of the scrotum with the absence of the testicles. p. 91.

"The penis being small and imperforate, may easily be mistaken for a clitoris; the division of the urethra may resemble the aperture of the vulva, especially when the scrotum is divided; while each division of the scrotum, whether it contains a testicle or not, represents with tolerable accuracy an external labium; and in some cases, the division of the penis forms two folds, which descend to the perineum, and may be mistaken for the nymphæ. In all these cases, the resemblance to the female organs is much more striking when the testicles remain in the abdomen. It is a very remarkable circumstance, that in a great proportion of those cases in which the original division of the male organs of generation continues to a greater or less extent, we likewise find other characteristics of the female sex, in these organs, as well as in the constitution and temperament of the individual."

We have already stated that if the growth of the fœtal organs were arrested at different periods, the monster would resemble various animals, according to the situation of the defective organ or tissue. If the heart, for instance, is wanting, the deformity resembles the natural state of the circulation in the dorsal insects,—if it is composed of only two cavities, it resembles the same organ in fishes and some of the molusca. The same may be said of other organs. It must be confessed, however, that we occasionally have deformities resembling in no respect any of the inferior animals. The cyclops is of this class. Here both eyes seem to have terminated in one, in consequence of the absence of the nasal bones, which is undoubtedly the result of an arrest of nutrition. In many instances the central eye has really been found, upon dissection, to be a double organ, situated in a single socket.

Our author next proceeds to the investigation of the second grand division of monstrosities,—that which is the result of an excess of nutrition. p. 104.

"Increase of size, when affecting the whole body, produces those monsters called giants: when confined to some particular organ, it is as often adventitious as congenital; in the latter case we have already seen that it frequently co-exists with atrophy of some other organ. The numerical increase of parts may be limited to a few organs, or may extend to every organ in the body; the parts affected are in general only doubled, hence, Meckel proposes to term this species of malformation, *duplication* of organs. In several cases of this kind, the duplication is so complete that it is highly probably, it proceeds from the junction or incorporation of two fœtuses."

The bones and muscles are more frequently increased in number than any other parts of the body. Such numerical variations however, can hardly be said to constitute a monstrosity. The vertebræ and ribs may be more numerous than common without any inconvenience whatever. If a supernumerary vertebra be only partly formed, then the lesion is of a serious character, but if it be complete the spinal chain is perfect, notwithstanding it consists of an unusual number of links. In the early part of this notice we referred to additional fingers, toes, etc. We shall say nothing further at present on this subject.

The external parts of the body are much more frequently increased, in number, than the internal. The existence of two tongues is occasionally found, but very seldom a double æsophagus or trachea. One of the Edinburgh Journals mentions a case of double duodenum, which terminated in a common pyloric orifice and there formed a kind of cul-de-sac. The writer of this review, once saw a full grown squirrel, which had two hearts, each distinctly formed, and entirely separate from the other. It was killed near Olivesburg, Richland county, Ohio, where the duplicate specimen was examined by several individuals. We do not know, that there is a similar case on record. Winslow relates a case of two hearts in a single thorax, but his specimen, besides being a cyclop, was otherwise imperfect. The trachea and æsophagus were wanting, thus presenting a specimen of the law of compensation mentioned by St. Hillaire.

Occasionally some of the organs of different sexes are found in the same individual. The Germans have collected a number of specimens of this kind, principally, if not entirely, confined to the genital organs. In no instance, however, have the entire organs of both sexes, been found, complete, in the same individual. In all cases the transformation exhibited nothing but fragments of the different organs. Indeed it is more than probable that the imagination has frequently had much to do, in rendering the duplication of the observer complete even thus far.

Monsters may sometimes be the result of the union of two fœtuses. The connexion may be more or less perfect, from the mere blending of a portion of the external integument, as in the case of the Siamese twins, to the duplication of many of the most important organs in a single skin. Sometimes the fragments of one fœtus, are really contained within the body of an other. A case of this kind undoubtedly occurred, a few years since, in the interior of Kentucky. A gentleman's male servant, after many years of ill health, discharged, per anus, at the age of sixteen, a number of bones, which presented all the appearances of those of a fœtus. Various specimens were sent to Dr. Drake, of this city, which we examined, and found among them a part of an os femoris, which, though very small, was tolerably perfect. After the osseous evacuations ceased, the boy enjoyed good health. This case was first ralated to us by our friend Dr. E. S. Clarkson, and since confirmed by Mr. Rannells, now a student of medicine in the Cincinnati College. Here the fœtus was undoubtedly formed within the boy, at the same time that his body was organized, but where it could have remained for sixteen years, cannot be ascertained. It was no doubt enveloped in a cyst, which eventually opened into either the colon or rectum. M. Dupuytren relates a similar case, and almost the only authentic one on record, where a cyst was found in the colon of a boy thirteen years of age, containing all the rudiments of a fœtus. In this case, however, the boy died, while in the one just related he lived and became healthy.

Occasionally a monstrosity presents itself to us with a double body and a single head, a double head and a single body, or a double head and body. All these preternatural growths appear to be the result of the union of two fœtuses during the early periods of utero-gestation. The ova, by some unknown means, have been placed in contact, and the vessels from each have shot into the other, and a deformity has been the result. In this case we must beg leave to differ in opinion from our able author, who thinks that this is altogether the result of an excess of nutrition. We think that this lesion should be charged to a defect in the nutritive process, for there certainly were two ova, and had the growth been perfect, duplicates would have been the result. The membranes peculiar to each fœtus, however, were wanting, the ova were placed in contact, and a blending of particles with a corresponding deformity was the consequence. When a monster is found with two heads or two bodies, the single body or head generally contains double organs. There are two stomachs and two alimentary canals, or two brains with or without a corresponding number of spinal marrows, and so of the other organs. Our author observes in relation to double monsters, p. 114,

"The last variety we shall enumerate, is that in which all the grand cavities are separate, at least externally; where, in short, there are two heads and two bodies. In this variety, the extremities occasionally present some anomalies; for instance, one of the lower extremities has been observed in merely a rudimentary state, attached as an appendix to the thigh of its fellow, which was well formed: while the leg belonging to this latter had seven toes on its foot.

"The junction of the fœtuses may take place at any point of the body; and, accordingly, they have been attached to each other; 1st, by the crown of the head, in the same right line; 2d, by the anterior portion of the thorax; 3d, by the anterior portion of the abdomen; 4th, by the dorsal spine; 5th, by the sacrum: 6th, by the nates, etc."

The last grand division of monstrosities to which our author directs our attention, is that which arises from a perversion of the *nisus formativus*, or nutritive force. This may produce monsters having neither an excessive nor imperfect development, but simply a transposition or misplacement of one or more of the several organs. Our author observes, p. 117.

"One of the most striking examples of this class is exhibited in the general transportation of the thoracic viscera; in which all those parts usually situated at the right side are found at the left, and *vice versa.* The heart affords a remarkable illustration of this transposition; its apex, instead of pointing towards the left, corresponds to the interval between the fifth and sixth ribs of the right side: its auricles and ventricles occupy a position exactly the reverse of their ordinary situation; and the aorta descends along the right side of the vertebral column. The left lung is divided into three lobes, whilst the right has only two. The pyloric orifice of the stomach is turned towards the left hypochondrium, which is occupied by the liver, the spleen being at the right side. The whole intestinal tube presents a similar transposition; thus the cœcum rests on the left iliac fossa, etc."

The primary cause of all these lesions is involved in entire obscurity. It may, perhaps, be some fault either of the ovum, or of its investing membranes. M. St. Hillaire is of the opinion that they are the result of unnatural adhesions, between the membranes and the fœtus, while it is in an embryotic state, and before many of the principal organs are formed. This may be the case in some instances, but it will not account for many of the most important alterations. Mechanical causes may occasionally derange the growth of the fœtus in utero. St. Hillaire found that he was enabled to arrest, and even alter the nutrition of the chick, at any period, by varnishing the shell in different places, or by shaking the egg at different times.

From all that has been advanced on the subject, it is more than probable that a large majority of monstrosities may be referred to vices of nutrition, rather than to diseases affecting the fœtus at any period. Indeed we cannot perceive how the number of the organs can be otherwise increased. Disease, either of the mother or the child, certainly could not form an additional number of fingers or toes; yet it is but a short time since this agent was supposed, even by the best writers, to produce every variation either in the number, or size, or place of the organs or tissues. Such a position, however, is so completely absurd, that it is only astonishing to us that it was not long since completely abandoned.

The absurd notions of the agency of the imagination of the mother, in the production of monstrosities, do not deserve a formal refutation.

It is probable, from the experiments of a distinguished naturalist, of Geneva, that the amount of nutriment, furnished the fœtus, exerts considerable influence over its development. The individual referred to, found that he could produce either males, or females, in bees, at pleasure, by simply placing the insect, at a very early period, in a cell containing more or less honey. W. W.

